# GPR120 modulates epileptic seizure and neuroinflammation mediated by NLRP3 inflammasome

**DOI:** 10.1186/s12974-022-02482-2

**Published:** 2022-05-27

**Authors:** Zhangjin Qin, Jiaqi Song, Aolei Lin, Wei Yang, Wenbo Zhang, Fuxin Zhong, Lihong Huang, Yang Lü, Weihua Yu

**Affiliations:** 1 Institute of Neuroscience, Chongqing Medical University, 1 Yixueyuan Road, Yuzhong District, Chongqing, 400016 China; 2 Department of Neurology, Chongqing Key Laboratory of Neurology, The First Affiliated Hospital of Chongqing Medical University, Chongqing, 400016 China; 3 Department of Integrated Traditional Chinese Medicine and Western Medicine, The First Affiliated Hospital of Chongqing Medical University, Chongqing, 400016 China; 4 Department of Geriatrics, The First Affiliated Hospital of Chongqing Medical University, 1 Youyi Road, Yuzhong District, Chongqing, 400016 China

**Keywords:** GPR120, Neuroinflammation, NLRP3 inflammasome, Epilepsy, Neuronal damage

## Abstract

**Background:**

The complex pathophysiology of epilepsy hampers the development of effective treatments. Although more than ten kinds of anti-seizures drugs (ASDs) have good effects on seizure control worldwide, about 30% of patients still display pharmacoresistance against ASDs. Neuroinflammation seems to play a crucial role in disease progression. G protein-coupled receptor 120 (GPR120) has been shown to negatively regulate inflammation and apoptosis. However, the role of GPR120 in epilepsy remains unclear. In this study, we aimed to explore the mechanism of GPR120 in epilepsy.

**Methods:**

Male adult C57BL/6 mice were intracranially injected with kainic acid (KA) to establish epilepsy model, and the adeno associated virus (AAV) was administered intracranially at 3 weeks before KA injection. VX765 was administered by intragastric administration at 30 min before KA induced and an equal dose administrated twice a day (10 a.m. and 4 p.m.) lasting 7 days until the mice were killed. Western blot analysis, immunofluorescence staining, video monitoring of seizure, LFP recording, Nissl staining were performed.

**Results:**

GPR120 was increased in both the hippocampus and cortex in the KA-induced model with temporal lobe epilepsy (TLE), and both were most highly expressed at 7 days after KA injection. Overexpression of GPR120 significantly alleviated epileptic activity, reduced neuronal death after status epilepticus (SE), downregulated the expression of IL-1β, IL-6, IL-18, and pyrin domain-containing protein 3 (NLRP3) inflammasome, whereas knockdown GPR120 showed the opposite effect. The effects of GPR120 knockdown were reversed by VX765 inhibition cysteinyl aspartate specific proteinase-1 (Caspase-1).

**Conclusion:**

GPR120 modulates epileptic seizure activity and affects neuronal survival in KA-induced mouse model of temporal lobe epilepsy. Furthermore, GPR120 regulated neuroinflammation in epileptic animals through NLRP3/Caspase-1/IL-1β signaling pathway.

## Introduction

Epilepsy is a common chronic neurological disorder often characterized by transient high synchronous abnormal discharges of neurons in the brain. Seizures not only affect patients’ quality of life, but also damage the brain, causing memory decline and cognitive disability. Although more than ten kinds of anti-seizures drugs (ASDs) have good effects on seizure control worldwide, about 30% of patients still display pharmacoresistance against ASDs. In recent research, the contributing role of neuroinflammation in epilepsy has become increasingly acknowledged. Therefore, understanding the mechanism of neuroinflammation in epilepsy is necessary so that it can provide further evidence to explore the pathogenesis of epilepsy and novel treatment targets.

G protein-coupled receptor 120 (GPR120), also known as free fatty acid receptor 4 (FFAR4), is activated by polyunsaturated fatty acids (PUFAs). It belongs to the G protein-coupled receptor family and is an important signaling molecule in cell function. When it binds to specific ligands, GPR120 can stimulate a variety of cellular responses via second messengers. GPR120 is widely expressed in a variety of tissues, which may contribute to its wide range of effects, involving in metabolic health, inflammation, and the dynamic regulation of immune processes [1–4]. It has been previously demonstrated that GPR120 is involved in energy control and regulation of energy metabolic efficiency in metabolic diseases such as obesity and diabetes [5, 6]. Some studies also indicate a relationship between diabetes and epilepsy. Insulin-related type 1 diabetes is positively associated with the risk of epilepsy [7], and shares genetic or autoimmune factors with epilepsy [8]. The incidence of epilepsy in the elderly is consistent with the incidence of obesity, diabetes and other chronic diseases. Besides, some ASDs also alter metabolic pathways and show anti-diabetic or obesity-ameliorating effects [9]. Therefore, GPR120 may be involved in epilepsy. This hypothesis is also supported by its involvement in neuroinflammation [10], since it is commonly activated in epileptogenic brain regions in humans and is clearly involved in animal models of epilepsy [11]. Activation of GPR120 inhibits the production of inflammatory cytokines. For example, activation of GPR120 inhibits the release of IL-1β, IL-6, and TNFα from renal tubular epithelial cells [12], and it can also reduce neuroinflammation and apoptosis in animal model of cerebral ischemia [11, 13]. This contributing effect of GPR120 in inflammation makes it a potential novel target in the regulating of epilepsy.

Recently, the activation of the pyrin domain-containing protein 3 (NLRP3) inflammasome has been found to be associated with various neurological disorders, such as Alzheimer’s disease [14], cerebrovascular disease [15, 16] and epilepsy [17]. NLRP3 inflammasome is composed of multiple proteins, mainly including NLRP3, apoptosis associated speck like protein containing a CARD (ASC), and cysteinyl aspartate specific proteinase-1 (Caspase-1). It can be activated when cells are subjected to specific stimuli, such as hypoxia or complement-mediated injury. Patients with mutations in NLRP3 gene have been found to secrete more proinflammatory cytokines, such as interleukin-1β (IL-1β) and interleukin-18 (IL-18) [18]. When NLRP3 is inhibited, the anti-epileptic and neuroprotective effects are observed after amygdala kindling induces status epilepticus [17]. These findings strongly suggest NLRP3 inflammasome participates in epilepsy through neuroinflammation.

Since the role of GPR120 in epilepsy has not been studied or fully illustrated, it is unclear whether its involvement in epilepsy is associated with the activation of NLRP3-mediated neuroinflammation, we set out this study to investigate the expression pattern of GPR120 in the brain of animal model of epilepsy and its specific role in regulating neuroinflammation and neuronal survival. We also aimed to explore the acting role of GPR120 in the activation of NLRP3 and downstream inflammation molecules in kainic acid (KA)-induced mouse model of temporal lobe epilepsy (TLE).

## Materials and methods

### Animals

Eight-week-old male mice (C57BL/6, the Laboratory Animal Center of Chongqing Medical University, Chongqing, China) weighing 25–30 g were housed in a standardized environment with a 12-h light/dark cycle and controlled temperature at 22 °C with food and water available ad libitum. All procedures were approved by the Commission of Chongqing Medical University for Ethics of Experiments on Animals and implemented in accordance with international standards.

### Hippocampus adeno associated virus (AAV) injection

AAV9-FFAR4-RNAi (GPR120-AAV-KD), AAV9-CON305 (Con-AAV-KD), AAV9-FFAR4 (GPR120-AAV-OE) and AAV9-CON323 (Con-AAV-OE) were designed and purchased from the Shanghai Genechem. Mice were anesthetized with sodium pentobarbital (50 mg/kg) and mounted onto the stereotaxic apparatus (RWD Life Science Co. Ltd., Shenzhen, China). Using a 5-μl syringe (Hamilton, Reno, NV), 0.5 μl of AAV9-FFAR4-RNAi or AAV9-FFAR4 was injected separately into the dentate gyrus (DG) and cornu ammonis subfield 1 (CA1) regions of right hippocampus at a rate of 0.05 μL/min. The coordinates are as follows: point 1 for DG, anteroposterior (AP), − 2.0 mm; mediolateral (ML), − 1.5 mm; dorsoventral (DV), − 2.0 mm; point 2 for CA1, AP: − 2.0 mm; ML: − 1.5 mm; DV: − 1.5 mm. The syringe was maintained in situ for an additional 10 min to minimize reflux along the injection trace. Then the same operation was repeated in the left hippocampus.

### KA model of epilepsy, video monitoring of seizure, and LFP recording

The chronic mouse model of spontaneous seizures induced by intrahippocampal administration of KA has been extensively described [19, 20]. KA model was established 3 weeks after AAV injection. Mice were anesthetized with sodium pentobarbital (50 mg/kg) and mounted onto the stereotaxic apparatus (RWD Life Science Co. Ltd., Shenzhen, China). Using a 0.5-μl syringe (Hamilton, Reno, NV), 1.0 nmol of KA (Sigma-Aldrich Co., St. Louis, USA) in 50 nl of saline was injected into the right hippocampus [AP: − 2.0 mm; ML: − 1.5 mm; DV: − 1.5 mm] over 3 min [20]. The syringe was maintained in situ for an additional 5 min to minimize reflux along the injection trace. Animals were randomly divided into 5 groups: mice without treatment group (Control group), mice with Con-AAV-KD and KA treatment group (Con-KD group), mice with GPR120-AAV-KD and KA treatment group (GPR120-AAV-KD group), mice with Con-AAV-OE and KA treatment group (Con-OE group), mice with GPR120-AAV-OE and KA treatment group (GPR120-AAV-OE group).

Two hours after KA injection, nonconvulsive SE was terminated using diazepam. Mice were placed under the video monitoring system to record the seizure of them for a month. The seizure grade was recorded according to the Racine’s scales as follow: stage 0, no response or behavior arrest; stage 1, chewing or facial twitches; stage 2, chewing and head nodding or wet dog shakes; stage 3, unilateral forelimb clonus; stage 4, bilateral forelimb clonus and rearing; stage 5, bilateral forelimb clonus, rearing and falling [21].

One month after SE induction, the local field potential (LFP) recording was performed for 2 h as previously described [9]. Briefly, the head of the awake mouse was fixed to minimize behavioral state–induced LFPs changes. LFPs were recorded using an MAP data acquisition system (Plexon, Dallas, TX). The signals were filtered (0.1–500 Hz), preamplified (1000 ×), and digitized at 4 kHz. The LFPs data were inspected using NeuroExplorer (Nex Technologies, Littleton, MA). A cluster of spontaneous paroxysmal discharges with a high-amplitude spike activity above 2 SDs from the baseline and a frequency above 1 Hz was defined as an seizure-like events (SLE, refers to the occurrence of epileptiform discharges in the brains of detected animals under EEG/LFP monitoring) if it lasted for 5 s or more.

### Immunofluorescence staining

Immunofluorescence was performed as previously described by our laboratory [22]. Briefly, tissue sections were fixed in 4% polyformaldehyde for 1 min, washed three times with phosphate-buffered saline (PBS), permeabilized with 0.4% Triton X-100 for 30 min, and blocked with goat serum working liquid (Wuhan Boster Biological Technology, Wuhan, China) for 1 h. The sections were then incubated overnight with mixed primary antibodies at 4 °C, washed in PBS to remove unbound primary antibodies, and incubated with secondary antibodies in the dark at RT for 1 h. The primary antibodies included rabbit anti-GPR120 (1:100; Affinity, China), mouse anti-NeuN antibody (1:100; Abcam, USA), mouse anti-GFAP antibody (1:100; SAB, China), and goat anti-Iba1 antibody (1:100; Abcam, USA). The fluorophore-conjugated secondary antibodies used were goat anti-mouse Alexa Fluor 650 (1:100; Abcam, USA), goat anti-rabbit Alexa Fluor 488 (1:100; Wuhan Boster Biological Technology, China), donkey anti-goat Alexa Fluor 549 (1:100; Wuhan Boster Biological Technology, China), and donkey anti-rabbit Alexa Fluor 488 (1:100; Wuhan Boster Biological Technology, China). Images were captured by confocal laser scanning microscopy (Leica, Wetzlar, Germany). The fluorescence intensity was analyzed using Image-Pro Plus 6.0, and colocalization analyses were performed using ZEISS.

### Nissl staining

Serial transverse sections made from brain tissue of mice embedded in a paraffin block were dewaxed, rehydrated, and immersed in 1% toluidine blue for 15 min. After washing in water, the sections were dehydrated in graded alcohols, cleared in xylene, and cover-slipped with neutral balsam. The stained sections were analyzed for residual neurons in CA1 and DG regions of hippocampus, and the number of surviving neurons was confirmed by the exhibition of Nissl substance, euchromatic nucleus, and nucleolus [23, 24]. From each brain tissue, approximately three sections were inspected, and all slides were assessed blindly with respect to treatment.

### Western blot

Hippocampal neurons and brain tissue samples were collected for Western blot analysis. Protein samples were separated by 10% SDS–polyacrylamide gel electrophoresis gels and electrotransferred onto 0.22-μm polyvinylidene difluoride membranes (Millipore, USA). The membranes were blocked with 5% nonfat dry milk in Tris-buffered saline with Tween (TBST) at RT for 1 h and then incubated with mouse anti-GPR120 (1:500; Santa Cruz, USA), mouse anti-Caspase-1 (1:500; Santa Cruz, USA), mouse anti-IL-1β (1:1000; CST, UK), mouse anti-IL-18 (1:500, Proteintech, China), rabbit anti-NLRP3(1:500; Proteintech, China), rabbit anti-ASC (Proteintech, China), rabbit anti-GAPDH (Proteintech, China) antibodies overnight at 4 °C. The blots were washed three times and incubated for 1 h with horseradish peroxidase HRP-conjugated anti-mouse or anti-rabbit secondary antibodies (1:5000; Proteintech) in TBST. The blots were then washed in TBST, and bands were visualized using an enhanced chemiluminescence system (Advansta, USA) and the ChemiDoc Touch Imaging System (Bio-Rad). Relative protein levels were determined by normalization to the GAPDH signal using Image lab software (Bio-Rad, CA, USA).

### Drug treatment

Animals were randomly divided into 3 groups: Con-AAV-KD epilepsy model with corn oil treatment group (KA group), AAV-KD epilepsy model with corn oil treatment group (KD + KA group), AAV-KD epilepsy model with VX765 treatment group (KD + KA + VX765 group).

VX765 powder was dissolved in corn oil with a final concentration of 1 mg/ml. These three groups of animals received intragastric administration of corn oil (0.2 ml per mouse), corn oil (0.2 ml per mouse), or VX765 (0.2 ml per mouse), respectively, 30 min before establishing epilepsy model and an equal dose administrated twice a day (10 a.m. and 4 p.m.) lasting 7 days until the mice were killed [25].

### Statistical analysis

The study was conducted by researchers following the principle of randomization and blinding. Experiments were performed at least in triplicate (for each biological replicate, *n* ≥ 3). All data were analyzed using the GraphPad Prism version 6.01 statistical package. Data were expressed mean ± SEM. Unpaired t test or ANOVA was used for statistical analysis, and significance at *P* < 0.05, *P* < 0.01 or *P* < 0.001 is indicated in graphs by one, two or three asterisks, respectively.

## Result

### GPR120 expression and distribution in the epileptic brain

**Fig. 1:**
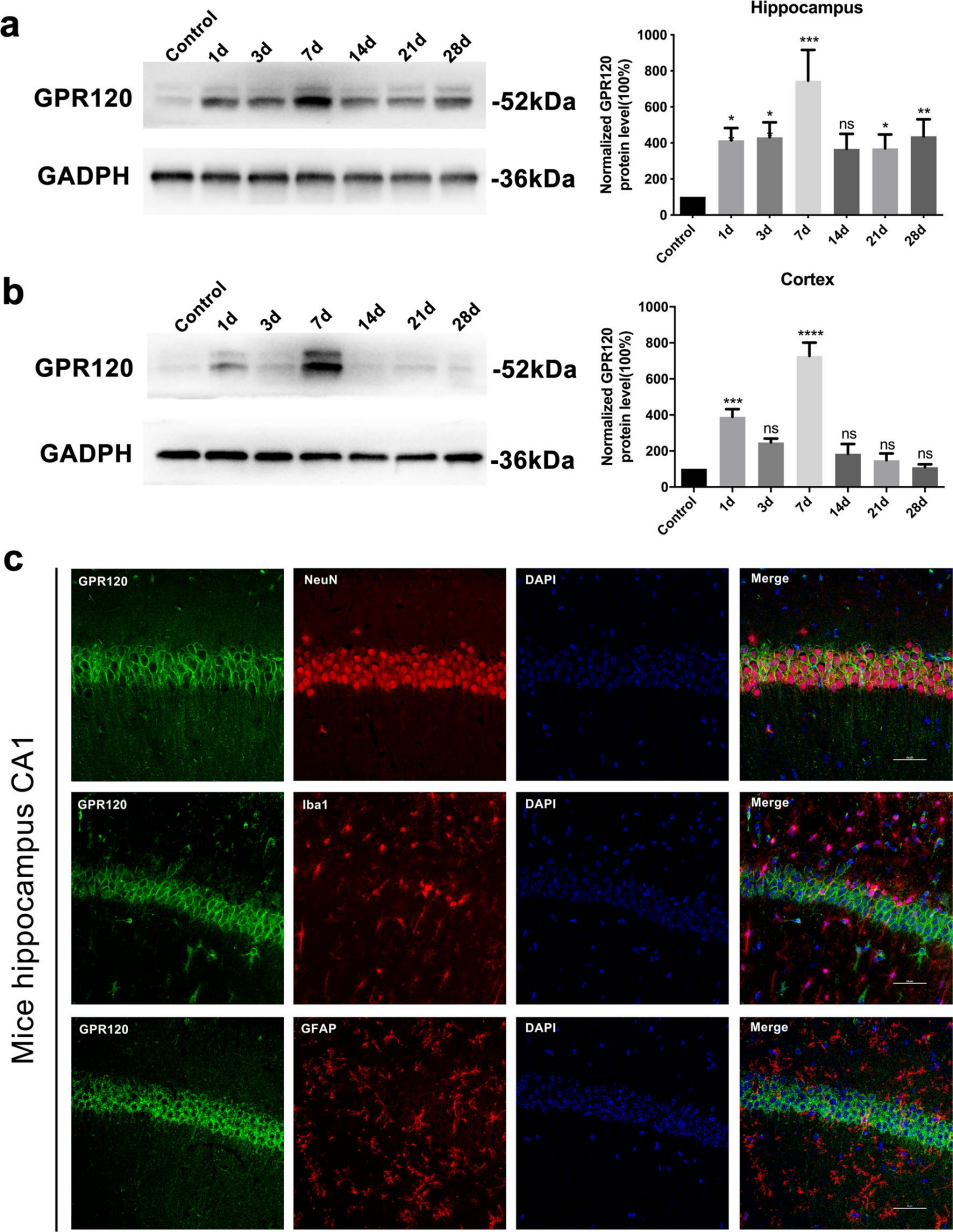
GPR120 expression and distribution in epilepsy. **a, b** Quantitative analysis of the normalized GPR120/GAPDH ratio, showing that the expression of GPR120 did increase in epilepsy mice model compare with normal control mice (*n* = 5 in each group; *P < 0.05 **P < 0.01, ***P < 0.001 versus Control group, Student’s *t* test). ns, not significant. **c** In hippocampal tissue from epilepsy mice, GPR120 colocalized with NeuN but not with Iba1 and GFAP (scale bar = 50 μm)

Previous studies have shown that GPR120 can be expressed in the brain of healthy primates and rodents [9, 26, 27], but its expression and distribution in brain regions closely associated with epilepsy, namely the hippocampus and cortex, are unknown. We first examined the expression levels of GPR120 by Western blot in a KA-induced TLE model. The KA-induced TLE model well recapitulated human TLE with typical hippocampal sclerosis [19, 28]. Bilateral hippocampi and ipsilateral (KA injected side) cortices of epileptic mice were obtained at 1, 3, 7, 14, 21, 28 days after intracranial KA injection. Compared with normal control group, GPR120 was increased in both the hippocampus and cortex in the KA-induced TLE model (*P* < 0.05), with a consistent trend, and both were most highly expressed at 7 days after KA injection (Fig. 1a, b) (*P* < 0.05).

**Fig. 2:**
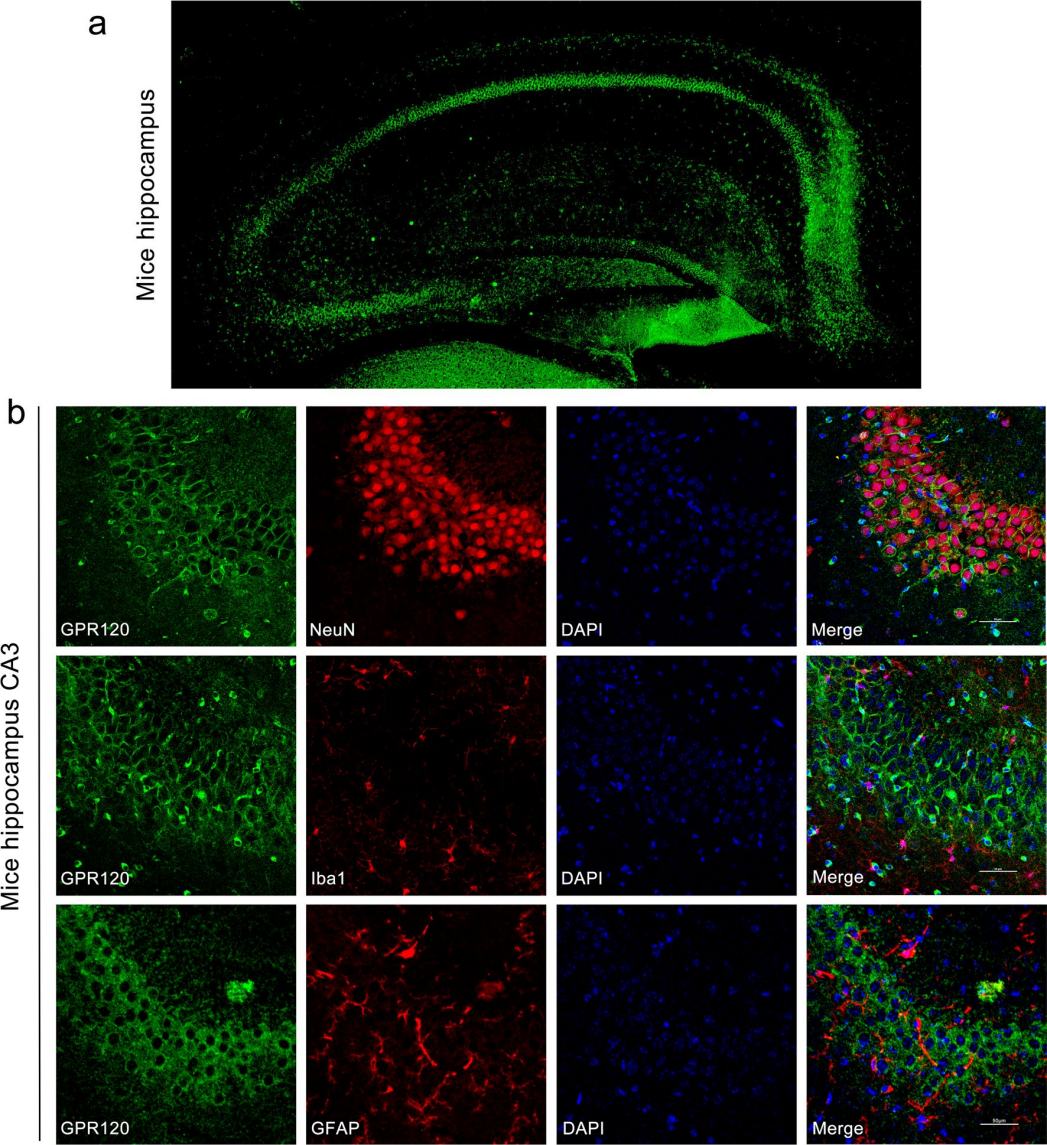
Immunofluorescent labeling of GPR120 in epileptic brain tissues. **a** GPR120 localization in the hippocampus. **b** In hippocampal tissue from epilepsy mice, GPR120 colocalized with NeuN but not with Iba1 and GFAP (scale bar = 50 μm)

**Fig. 3:**
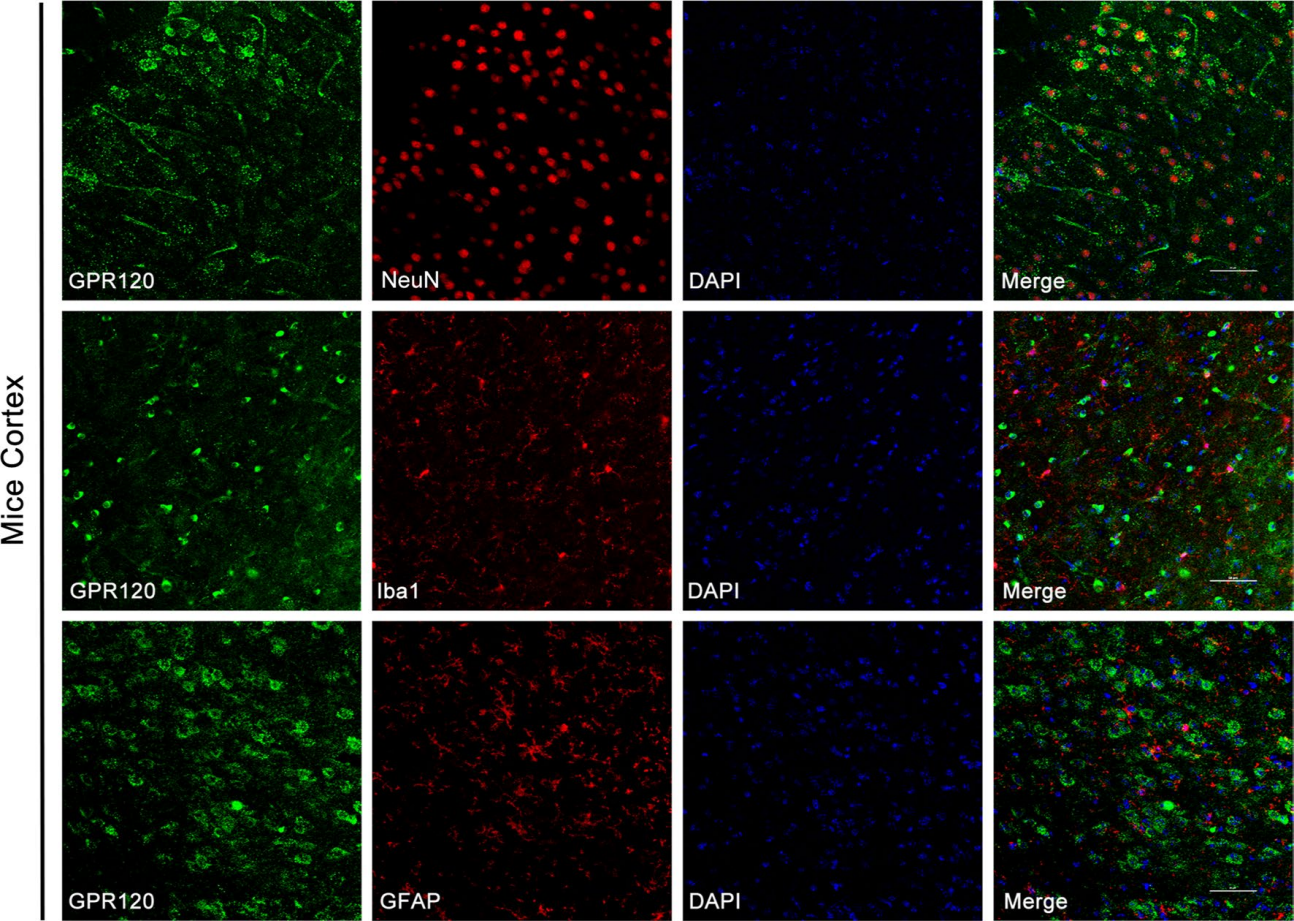
Immunofluorescent labeling of GPR120 in epileptic brain cortex tissues. In cortex tissue from epilepsy mice, GPR120 colocalized with NeuN but not with Iba1 and GFAP (scale bar = 50 μm)

To further verify the distribution of GPR120 in epileptic tissue, we next established an epileptic model by unilateral intrahippocampal injection of KA and obtained brain tissue from epileptic mice at 7 days after injection, when GPR120 expressed peaks. Immunofluorescence was used to examine the distribution of GPR120 in the hippocampus and cortex of epileptic mice, and we found that GPR120 was highly expressed in the hippocampal CA1 and CA3 regions of epileptic mice, but the expression was not significant in the DG region (Fig. 2a). To further determine the more precise localization of GPR120 in epilepsy, we examined the cellular localization of GPR120 in epileptic brain tissue by immunofluorescence double antibody labeling. GPR120 colocalized with neuronal nuclei (NeuN; a marker of neuron) but not with glial fibrillary acidic protein (GFAP; a marker of astrocyte) and ionized calcium binding adapter molecule 1 (Iba1; a marker of microglia) in the hippocampus of the KA-induced TLE mouse model, showing that GPR120 is expressed mainly in neurons but not in astrocytes and microglia (Figs. 1c, 2b and 3). To further demonstrate cellular colocalization characteristics of GPR120, we have computed the overlap percentage of GPR120/NeuN colocalization. The percentage of overlap (Merge-positive cells/GPR120-positive cells) was 89% (88.82 ± 0.34) for the CA1 region, 96% (95.51 ± 0.32) for the CA3 region of the hippocampus, and 80% (79.87 ± 0.18) for the cortex.

Taken together, GPR120 expression is increased in the epileptic brain and colocalizes with neurons in the epilepsy-related hippocampal CA1 and CA3 regions, suggesting that GPR120 may be involved in epileptic activity.

### GPR120 modulates epileptic seizure activity and affects neuronal death after SE

**Fig. 4:**
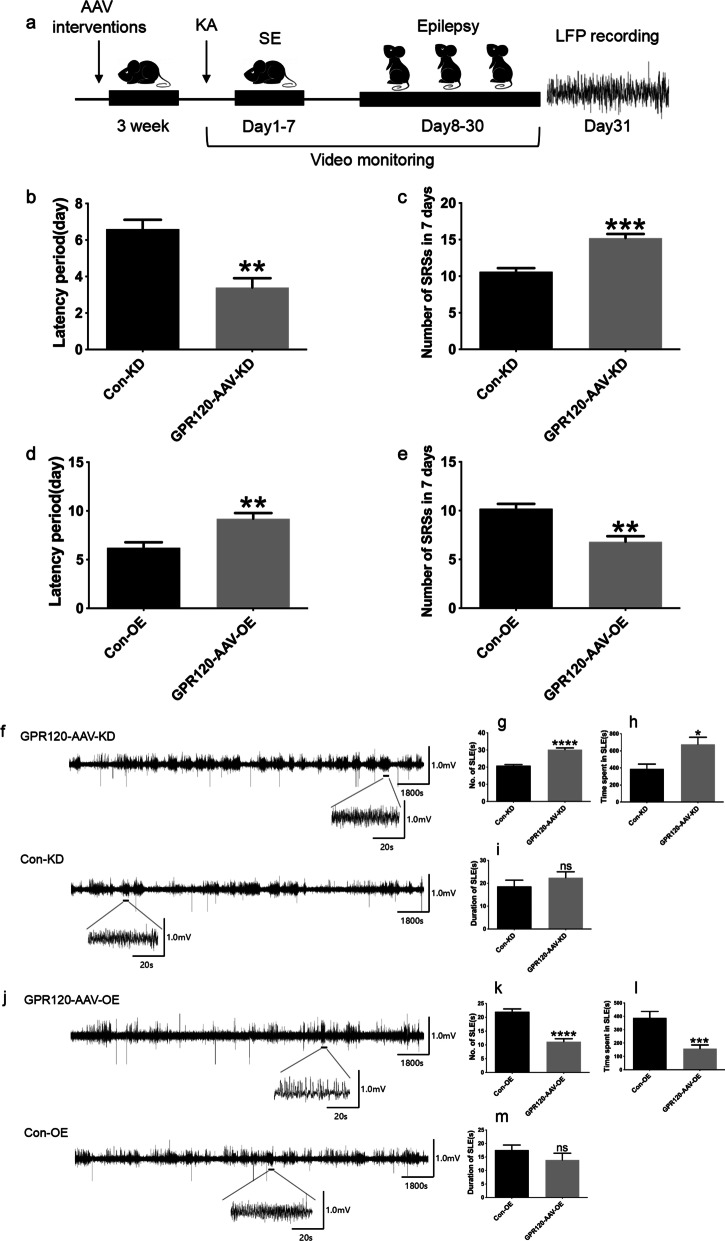
GPR120 modulates epileptic seizure activity. **a** Graphic representation of the experimental timeline in the KA experiment. Mice were inject with GPR120-AAV-KD, CON-AAV-KD, GPR120-AAV-OE, CON-AAV-OE 3 weeks before KA injection (*n* = 6 in each group). **b**, **d** At the beginning of video monitoring, the latency period were reduce in the GPR120-AAV-KD group and increased in the GPR120-AAV-OE group compared with the Con-KD group and Con-OE group. **c**, **e** At the 24–30 days after KA injection in video monitoring, the Number of SRSs was increased in the GPR120-AAV-KD group and reduced in the GPR120-AAV-OE group compared with the Con-KD group and Con-OE group. **f**, **j** Representative LFPs in the four groups. **g**, **k** During 30 min, the number of SLEs and was increased in the GPR120-AAV-KD group and reduced in the GPR120-AAV-OE group compared with the Con-KD group and Con-OE group. **h**, **l** The total time spent in SLEs during the 30 min was increased in the GPR120-AAV-KD group and reduced in the GPR120-AAV-OE group compared with the Con-KD group and Con-OE group. **i**, **m** The duration of SLEs was not significantly changed between the groups. For the analysis, *n* = 6 for each group. Error bars represent the means ± SEM; *P < 0.05, **P < 0.01, ***P < 0.001 versus the Con-KD or Con-OE group, Student’s *t* test. *ns* not significant

Changes in the expression of GPR120 in epilepsy could be either a phenomenon caused after epileptogenesis or a cause of seizures. Therefore, GPR120 knockdown virus (GPR120-AAV-KD) and GPR120 overexpression virus (GPR120-AAV-OE) can be intracranially injected CA1 and DG regions of bilateral hippocampi of mice to investigate the effect of GPR120 on regulating epileptic activity in the KA-induced TLE mice model. Before injecting, western blotting was used to detect the effect of GPR120 knockdown and overexpression. Compared to the sham-operated groups, the knockdown efficiency of GPR120 was 58% and overexpression efficiency was 166%. And then mice in the GPR120 knockdown virus group (KD group) and GPR120 overexpression virus group (OE group) were injected with GPR120-AAV-KD or GPR120-AAV-OE in the CA1 and DG regions of bilateral hippocampi, respectively. Their corresponding sham-operated groups (Con-KD group and Con-OE group) were injected with empty vector virus (Con-AAV-KD and Con-AAV-OE), respectively. Three weeks later after its successful and stable production of infection, the mice TLE model were established by intracranial injection of KA. Then, the behavior of mice was monitored continuously for 1 month, and the time from the start of KA injection until the occurrence of the first grade IV–V episode was recorded as the onset latency and episode that greater than grade IV were considered as spontaneous and recurrent seizures (SRSs, refers to spontaneous seizures that are visible to the naked eye under behavioral monitoring). The seizure frequency was recorded 24–30 days after KA injection. It was found that mice in the KD group exhibited a shorter latencies to seizure onset and increased seizure frequency in 7 days compared with the Con-KD group (3.40 ± 0.51 days of latency for KD group versus 6.60 ± 0.51 days of latency for Con-KD group; 15.20 ± 0.58 of frequency for KD group versus 10.60 ± 0.51 of frequency for Con-KD group) (Fig. 4b, c). In contrast, mice in the OE group exhibited prolonged seizure latencies and decreased seizure frequency compared with mice in the Con-OE group (9.20 ± 0.58 days of latency for OE group versus 6.20 ± 0.58 days of latency for Con-OE group; 6.80 ± 0.58 of frequency for OE group versus 10.20 ± 0.50 of frequency for Con-OE group) (Fig. 4d, e). Seizure activity was recorded using LFPs 1 month after SE induction. Consistent with other studies, frequent, repetitive SLEs were observed in all mice (Fig. 4). We analyzed the SLEs for a period of 30 min and found that the duration did not differ among the groups (22.44 ± 2.60 s for KD group versus 18.61 ± 2.744 s for Con-KD group; 13.91 ± 2.49 s for OE group versus 17.58 ± 1.86 s for Con-OE group) (Fig. 4i, m). However, compared with the Con-KD group, GPR120-AAV-KD increased the number of SLEs and the total time spent in SLE during a 30-min period (30.18 ± 0.99 of the SLEs number for KD group versus 20.80 ± 0.66 of the SLEs number for Con-KD group; 677.90 ± 80.41 s of the total time for KD group versus 387.00 ± 57.96 s of the total time for Con-KD group) (Fig. 4g, h). GPR120-AAV-OE had the opposite effect (11.18 ± 1.05 of the SLEs number for OE group versus 22.00 ± 1.05 of the SLEs number for Con-OE group; 157.20 ± 28.42 s of the total time for OE group versus 388.60 ± 47.26 s of the total time for Con-OE group) (Fig. 4k, l).

**Fig. 5:**
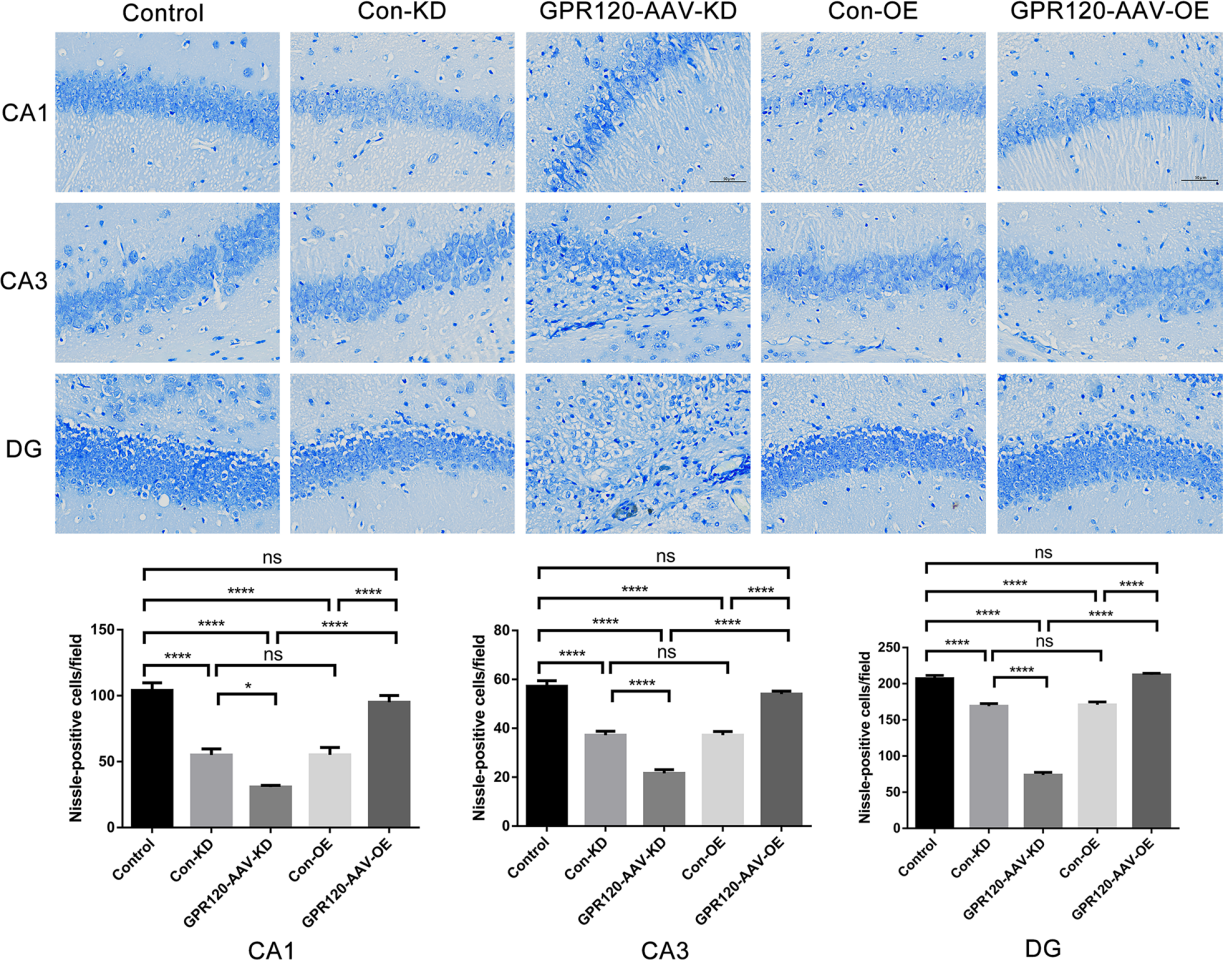
GPR120 affects neuronal death after SE. GPR120-AAV-KD markedly decreased the number of surviving cells after SE, whereas GPR120-AAV-OE showed the opposite effect. There were not statistically significant between GPR120-AAV-OE group and Control group in CA1, CA3 and DG regions (scale bar = 50 μm). For the analysis, *n* = 3 for each group. Error bars represent the means ± SEM; *P < 0.05, **P < 0.01, ***P < 0.001 versus the Control, Con-KD, Con-OE, GPR120-AAV-KD or GPR120-AAV-OE group, one-way analysis of variance (ANOVA), followed by Tukey's test. ns, not significant

To further explore the effect of GPR120 on neuronal survival, Nissl staining was performed at 1 month after SE induction. We found that the number of neurons in the CA1, CA3, and DG regions of the hippocampus was significantly decreased in the mice of KD group compared with the mice in Control group and Con-KD group (30.80 ± 1.24 of CA1 for KD group versus 104.00 ± 5.86 of CA1 for Control group and 55.00 ± 4.73 of CA1 for Con-KD group. 21.60 ± 1.50 of CA3 for KD group versus 57.20 ± 2.31 of CA3 for Control group and 37.20 ± 1.66 of CA3 for Con-KD group. 73.80 ± 3.77 of CA3 for KD group versus 207.2 ± 4.43 of DG for Control group and 169.00 ± 3.65 of DG for Con-KD group) (Fig. 5). However, the number of neurons in the CA1, CA3, and DG regions of the hippocampus in the mice of OE group was increased compared to the Con-OE groups (95.00 ± 5.21 of CA1 for OE group versus 55.20 ± 5.67 of CA1 for Con-OE group. 54.00 ± 1.23 of CA3 for OE group versus 37.20 ± 1.53 of CA3 for Con-OE group. 212.40 ± 2.14 of DG for OE group versus 171.00 ± 3.82 of DG for Con-OE group) (Fig. 5). There was no statistical significance between OE group and Control group in CA1, CA3 and DG regions (95.00 ± 5.21 of CA1 for OE group versus 104.00 ± 5.86 of CA1 for Control group. 54.00 ± 1.23 of CA3 for OE group versus 57.20 ± 2.31 of CA3 for Control group. 212.40 ± 2.14 of DG for OE group versus 207.20 ± 4.43 of DG for Control group). This indicated that knockdown of GPR120 could aggravate neuron loss in epileptic mouse, while overexpression of GPR120 could alleviate status epilepticus-induced neuron loss to some extent.

In summary, knockdown of GPR120 could exacerbate seizures, increase the frequency and total time of seizures, and aggravate the loss of neurons, while overexpression of GPR120 produced results opposite to knockdown of GPR120 in terms of reduced seizure frequency and total time, reduced neuronal loss after SE, and alleviated seizures.

### GPR120 regulates neuroinflammation in epilepsy

An increasing number of studies have shown that neuroinflammation plays an important role in the generation and exacerbation of epilepsy [29–31]. Cytokines play a central role in the immune system and inflammatory responses and are key mediators of inflammation. Proinflammatory cytokines (IL-1β, IL-6, and IL-18) contribute to the pathophysiology of epilepsy through several pathways: modulating glutamatergic transmission [32], enhancing N-methyl-D-aspartate receptor function by activation of SRC tyrosine kinase [33], and altering GABAergic neurotransmission [34]. Therefore, we used the cytokines IL-1β, IL-18, and IL-6, which are all commonly found in neuroinflammation as inflammatory markers to explore the effect of treatment with GPR120-AAV-KD and GPR120-AAV-OE on neuroinflammation in epilepsy.

**Fig. 6:**
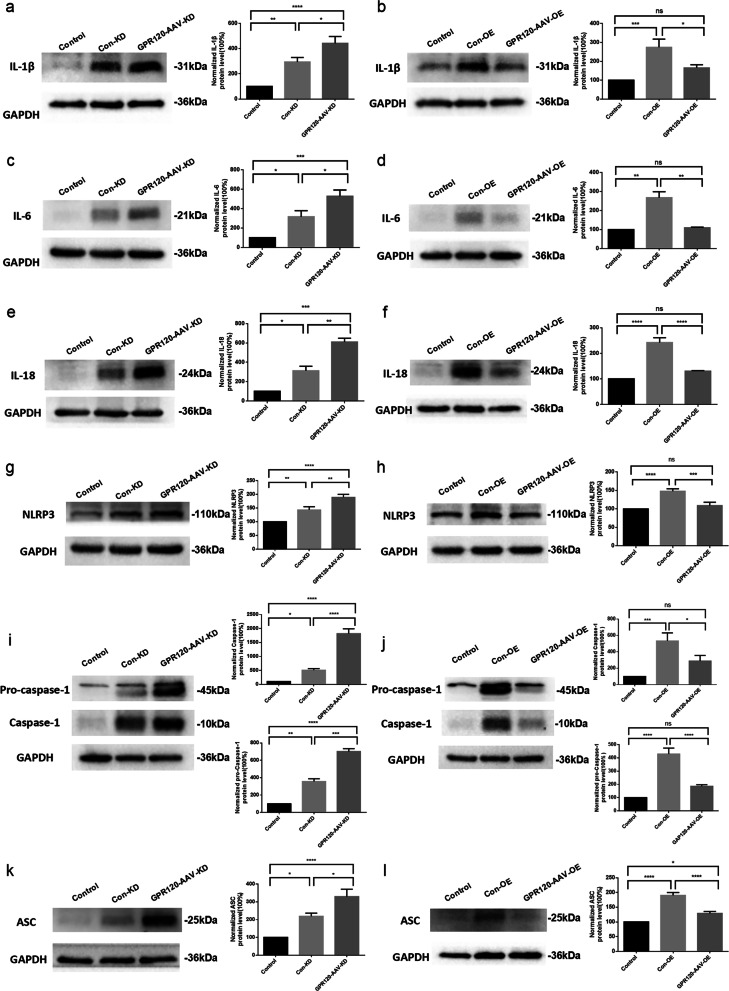
GPR120 affects proinflammatory cytokine production and NLRP3 inflammasome expression after SE. **a, c, e** The protein levels of IL-1β, IL-6 and IL-18 in hippocampal were increased after SE, and further increased by GPR120-AAV-KD. **b, d, f** SE-induced increases in IL-1β, IL-6 and IL-18 protein levels were markedly attenuated by GPR120-AAV-OE. **g, i, k** Increased the hippocampal protein levels of NLRP3, Caspase-1 and ASC after SE were markedly further increased by GPR120-AAV-KD. **h**, **j**, **l** Increased hippocampal protein levels of NLRP3, Caspase-1 and ASC after SE were markedly attenuated by GPR120-AAV-OE. For the analysis, *n* = 6 for each group. Error bars represent the means ± SEM; *P < 0.05, **P < 0.01, ***P < 0.001 versus the Control, Con-KD or Con-OE group, one-way analysis of variance (ANOVA), followed by Tukey’s test. *ns* not significant

Western blotting studies for proinflammatory cytokines were performed at 7 days after KA injection based on the peak expression of GPR120. Compared with the normal control group (control group), the levels of cytokines IL-1 β, IL-18, and IL-6 were significantly increased in the hippocampus of mice in the TLE model group intracranially injected with Con-AAV-KD and Con-AAV-OE (Con-KD and Con-OE groups) (Fig. 6a–f). The levels of the cytokines IL-1β, IL-18, and IL-6 were all significantly increased in the hippocampus of the TLE model mice intracranially injected with GPR120-AAV-KD (KD group) compared with the control and Con-KD groups (Fig. 6a, c, e). In contrast, the levels of cytokines IL-1β, IL-18, and IL-6 in the hippocampus of mice in the TLE model group intracranially injected with GPR120-AAV-OE (OE group) were significantly decreased compared with the Con-OE group. There was no statistical significance between OE group and control group (Fig. 6b, d, f).

In this part of the study, our results showed that knockdown of GPR120 in epilepsy model could cause elevated expression of inflammatory factors in the hippocampus of mice, and overexpression of GPR120 could result in decreased expression of inflammatory factors in the hippocampus of epileptic mice. These results suggest that GPR120 could regulate neuroinflammation in epilepsy.

### GPR120 regulates NLRP3 inflammasome expression

The NLRP3 inflammasome is an inflammasome complex composed of three proteins, NLRP3, ASC, and Caspase-1, and its activation can lead to Caspase-1 proteolytic activation and increase secretion of proinflammatory cytokines IL-1β and IL-18. Activation of NLRP3 inflammasome is closely associated with neuronal damage in multiple neurological diseases [35, 36]. Thus, we further explored the effect of treatment of GPR120 overexpression and knockdown virus on NLRP3 inflammasome expression. Western blotting studies for NLRP3 inflammasomes were performed at 7 days after KA injection based on the peak expression of GPR120.

Our study found that, similar to previous results, the levels of NLRP3, ASC, and Caspase-1 in the hippocampus were significantly increased in the Con-KD and Con-OE mice compared with the control mice. Compared with the control and Con-KD groups, NLRP3, ASC, and Caspase-1 levels were significantly increased in the hippocampus of mice in KD group (Fig. 6g, i, k). However, the levels of NLRP3, ASC, and Caspase-1 in the hippocampus were significantly decreased in the OE group compared with the Con-OE group. And there was no statistical significance between the OE group and control group (Fig. 6h, j, l).

Our results showed that both overexpression and knockdown of GPR120 led to NLRP3, ASC and Caspase-1 expression changes in the hippocampus of epileptic mice, and the trend of both changes was consistent with the changes in inflammatory factors described earlier, which indicated that GPR120 could regulate NLRP3 inflammasome complex activation.

### GPR120 affects epileptic seizure activity, neuroinflammation and neuronal death in epilepsy by regulating the activation of NLRP3 inflammasome

**Fig. 7:**
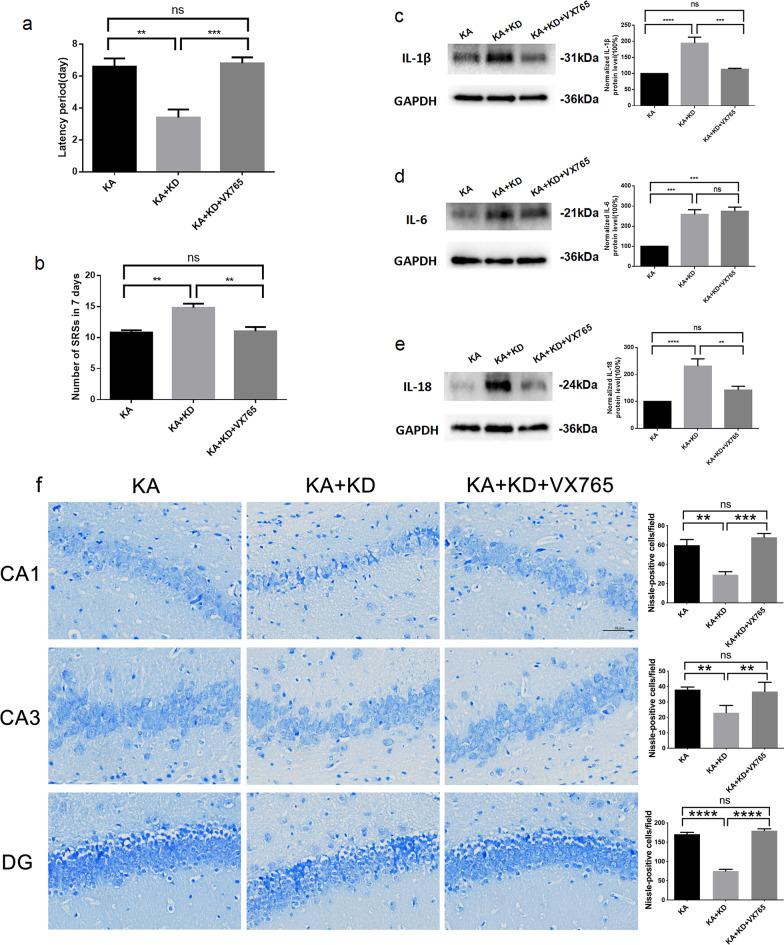
Caspase-1 inhibitor VX765 rescues the epileptic seizure activity, proinflammatory cytokine production and neuronal death caused by GPR120-AAV-KD. **a, b** Reduced latency period caused by GPR120-AAV-KD in epilepsy was rescued by VX765. **c, d** GPR120-AAV-KD induced increases in IL-1β, and IL-18 protein levels were markedly blocked by VX765. **e** VX765 has less effect on the protein level of IL-6. **f** The reduced number of surviving cells after SE attributed to GPR120-AAV-KD were rescued by VX765 (scale bar = 50 μm). For the analysis, *n* = 6 for each group. Error bars represent the means ± SEM; *P < 0.05, **P < 0.01, ***P < 0.001 versus the KA, KA + KD or KA + KD + VX765 group, one-way analysis of variance (ANOVA), followed by Tukey’s test. *ns* not significant

VX765 is a specific inhibitor of Caspase-1. In this study, epileptic mice treated with GPR120-AAV-KD partially were intragastrically administrated with VX765 (KD + KA + VX765 group), and another were intragastrically administrated with the same volume of corn oil solvent (KD + KA group). Epileptic mice injected with Con-AAV-KD were also intragastrically administrated with the same volume of corn oil solvent (KA group). As in the previous experiment, the seizure latency and frequency were recorded. It was found that mice in the KD + KA + VX765 group exhibited prolonged seizure latencies and decreased seizure frequency compared with mice in the KD + KA group, and were not statistically significant compared with those in the KA group (6.80 ± 0.37 days of latency for KD + KA + VX765 group versus 3.40 ± 0.51 of latency for KD + KA group and 6.60 ± 0.51 of latency for KA group 11.00 ± 0.71 of frequency for KD + KA + VX765 group versus 14.80 ± 0.66 of frequency for KD + KA group and 10.80 ± 0.37 of latency for KA group) (Fig. 7a, b).

To explore the mechanism of GPR120 affecting epileptic seizures, the levels of inflammatory factors were detected by western blot. We found that the levels of IL-1β and IL-18 in the brain of mice in the KD + KA + VX765 group were significantly reduced compared with those in the KD group and were not statistically significant compared with those in the KA group (Fig. 7c, e); whereas VX765 had less effect on hippocampal IL-6 levels in mice treated with GPR120-AAV-KD (Fig. 7d). These results suggest that VX765 can rescue the neuroinflammation caused by GPR120-AAV-KD in epilepsy, which further illustrated that GPR120-AAV-KD may cause neuroinflammation in epilepsy by promoting the expression of NLRP3 and thus activating Caspase-1.

As previously mentioned, GPR120 knockdown can cause increased neuronal damage, so we further investigated the effect of VX765 on neuronal damage. The results of Nissl staining showed that the number of surviving neurons in the CA1, CA3, and DG regions of the hippocampus was significantly increased in the KD + KA + VX765 group compared with the KD + KA group, and there was no statistical difference compared with the KA group (68.00 ± 3.94 of CA1 for KD + KA + VX765 group versus 29.25 ± 3.12 of CA1 for KD + KA group and 59.75 ± 5.98 of CA1 for KA group. 36.85 ± 3.01 of CA3 for KD + KA + VX765 group versus 23.00 ± 2.38 of CA3 for KD + KA group and 38.00 ± 1.68 of CA1 for KA group. 179.80 ± 4.79 of DG for KD + KA + VX765 group versus 75.00 ± 4.51 of DG for KD + KA group and 170.50 ± 4.87 of CA1 for KA group) (Fig. 7f).

The findings in this section show that VX765 can rescue the neuroinflammation and neurotoxicity caused by GPR120-AAV-KD in the hippocampus of epileptic mice, indicating that GPR120 could regulate neuroinflammation in epilepsy which in turn affect neuronal damage through the activation of NLRP3 inflammasome complex.

## Discussion

In the present study, we firstly find GPR120 to be upregulated in brain tissue of TLE mice model, and we report a novel finding that GPR120 modulates epileptic seizure activity and affects neuronal survival in KA-induced mouse model of temporal lobe epilepsy. Furthermore, GPR120 can regulate neuroinflammation in epileptic animal model through NLRP3/Caspase-1/IL-1β signaling pathway.

In the hippocampus of epileptic mouse model, GPR120 was highly expressed in the pyramidal cell layer of CA1 and CA3 regions, which are the main regions of epileptic pathological alterations [37], and colocalized with neurons but not in astrocytes or microglia. Immunohistochemical labeling of human temporal lobe tissue adjacent to the sclerous hippocampus also showed GPR120 to be expressed exclusively in neurons. This information on localization of GPR120 was different from previous reports. Indeed, GPR120 may express in neurons, microglia and astrocytes to play different roles based on different pathological diseases, including injury, cerebral ischemic disease, hepatic injury [13, 38]. However, the pathological feature of epilepsy is the abnormal discharge of neurons, while the excessive energy consumption by neurons upon discharge is also a kind of depletion and stimulation on the neurons themselves, this kind of stimulation may also be produced by the neuron interiorly and then spread to other areas of the brain. GPR120 in the present study is mainly localized in the CA1 and CA3 pyramidal neurons, which respond to the pathological changes in epilepsy. Therefore, the role of GPR120 works via neurons in epilepsy.

The inflammatory response in the nervous system is complex. Although microglia play a key role in the inflammatory response of the central nervous system, but microglia are not the only cells which can cause the inflammatory response. Michoud et al. study showed that direct stimulation of neurons using optogenetics without additional damage can also lead to neuroinflammation [39]. Hua et al. also found that activation of NLRP3 and caspase-1 in neurons can also lead to neuroinflammation [40]. NLRP3 has been found to be significantly elevated in neurons [41] and microglia [42] in the epileptic brain, which is mainly stimulated by intracellular environment and cell membrane receptor signals [43]. Moreover, GPR120 is a membrane receptor that can be internalized into cells to regulate inflammatory factors under disease conditions [44]. When inflammation occurs, interaction of GPR120 and NLRP3 evokes internalization of GPR120 from the cell surface into internal cellular compartments, exerting anti-inflammatory effects [41]. However, such anti-inflammation of GPR120 is still not enough to compensate inflammatory damage in epilepsy; therefore, exogenous GPR120 is necessary for seizure alleviation. Our study shows that overexpression of GPR120 decreases the expression of NLRP3, and inflammatory cytokines are further suppressed, including IL-6, IL-18 and IL-1β, neuroinflammation is thus attenuated enough and seizures improve. Conversely, knockdown of GPR120 increases NLRP3-associated neuroinflammation, seizures aggravate. Meanwhile, seizures can be rescued by using caspase-1 (downstream effector molecules of NLRP3) inhibitors, further proving that GPR120 regulates epilepsy via NLRP3 inflammation. Our study shows that GPR120/NLRP3 pathway may play a role in neuroinflammation via internalization in neurons.

The pathophysiological changes of a disease include pathogenic insults that generate and aggravate its progress and protective modulations that alleviate this progression. In epilepsy, therefore, enhancing protective factors may also be a good strategy for suppressing seizure activity. In this study, we demonstrated the important role of GPR120 in modulating epileptic activity in KA-induced epilepsy model. Both frequency and overall duration of epileptic seizures were decreased after overexpressing GPR120, along with increased neuronal survival and decreased neuroinflammation, which indicated a compensatory and protective effect of GPR120 in epilepsy and verified our hypothesis that activation of GPR120 emitted anti-epileptic effect. Even though the expression of GPR120 has been increased in KA-induced epilepsy model, it is still not enough to exert anti-inflammatory effects. This is consistent with the results from a previous study on cerebral ischemia reporting that GPR120 was increased in cerebral ischemia models, and GPR120 was activated by DHA to produce anti-inflammatory effects [13]. Many studies have also shown that GPR120 is an important protective factor in the nervous system, and GLP-1 secretion, stimulated by intestinal GPR120, may remotely contributed to suppress PGD2-microglia-provoked neuroinflammation in the hippocampus [45]. However, its regulatory mechanism in epilepsy is still unclear.

Rodents with spontaneous seizures that mimic the features of human epilepsy can be used to study pathogenetic mechanisms and potential therapeutic targets of epilepsy. The cross-validation of animal and human findings adds considerable value to epilepsy research because it contributes to the deeper understanding of the mechanisms of epileptogenesis and ictogenesis [46]. Among these mechanisms, neuroinflammatory pathways are known to contribute to development and progression of the disease and could be targets for disease-modifying therapies that are beneficial in epilepsy with a range of aetiologies [11]. A large number of studies have shown that GPR120 can reduce the release of proinflammatory cytokines and participate in the inflammatory response of different tissues [6, 12, 13, 36, 47]. Therefore, we focused on the effects of GPR120 on neuroinflammation in epilepsy. We found that GPR120 activation and inhibition led to a decrease and increase in levels of proinflammatory cytokines IL-1β, IL-6, and IL-18, respectively. These observations are consistent with findings showing that GPR120 activation can downregulate TNF-α-related inflammatory responses [10], reduce energy metabolism efficiency and regulate obesity-related inflammatory responses in the hypothalamus [6]. Since neuroinflammation is not only a consequence of seizures or brain neuropathology, but also a mechanism involved in their generation [11], exploring the pathway of GPR120 that governs neuroinflammation in epilepsy has raised considerations for its importance.

The NLRP3 inflammasome is a protein complex that promotes the release of inflammatory factors in response to infection or tissue injury and is known to be associated with many nervous system diseases, such as Alzheimer’s disease (AD), depression and epilepsy [39–41]. The role of NLRP3 in neuroinflammation in combination with its broad range of mediators indicates that GPR120 might regulate neuroinflammation and manifest its protective effect in epilepsy through NLRP3-dependent signaling pathway. In this study, we found that GPR120 activation and inhibition led to a decrease and increase in levels of NLRP3, ASC and Caspase-1, respectively, as well as the corresponding changes of downstream proinflammatory cytokine IL-1β and IL-18. This suggests that GPR120 may act as a ligand of NLRP3 to regulate the activation of NLRP3 and the changes of its downstream inflammatory factors.

Given the possible impact of GPR120 on epilepsy via other signaling pathways, we further add the inhibitor of Caspase-1 on the basis of GPR120 knockdown, trying to verify whether inhibiting the activation of NLRP3 can rescue the impact of GPR120 knockdown on epilepsy. Our data indicated that Caspase-1 inhibitor could significantly rescue the increased level of IL-1β and IL-18 caused by GPR120 knockdown, but had little effect on the change of IL-6. Since IL-1β and IL-18 are signaling pathways downstream of NLRP3, our data can indirectly prove that GPR120, as a ligand of NLRP3, regulates the activation of NLRP3 and induces neuroinflammation in epilepsy. Finally, our data showed that Caspase-1 inhibitors can save neuronal death caused by GPR120 knockdown, reduce the number of seizures and increase the latency of seizures. This indicated that GPR120 also plays a role in the regulation of epilepsy through NLRP3. These results confirmed that regulation of GPR120 suppressing NLRP3-mediated neuroinflammation is a possible mechanism by which GPR120 regulates epileptic activity and neuronal survival.

## Conclusion

In conclusion, our findings demonstrate that GPR120 activation can attenuate epileptic seizures, increase neuronal survival and suppress neuroinflammation by inhibiting NLRP3/Caspase-1/IL-1β signaling pathway in mouse epileptic model, providing a novel anti-epileptic target. The neuroprotective mechanism of GPR120 action was preliminarily revealed to be the regulation of NLRP3 inflammasome, although further mechanisms remain to be explored.

## Data Availability

The data sets generated for this study are available on request to the corresponding authors.

## Abbreviations

GPR120: G protein-coupled receptor 120
SE: Status epilepticus
NLRP3: NOD-like receptor pyrin domain-containing protein 3
ASDs: Anti-seizure drugs
FFAR4: Free fatty acid receptor 4
PUFAs: Polyunsaturated fatty acids
ASC: Apoptosis associated speck like protein containing a CARD
Caspase-1: Cysteinyl aspartate specific proteinase-1
IL-1β: Interleukin-1β
IL-18: Interleukin-18
IL-6: Interleukin-6
KA: Kainic acid
TLE: Temporal lobe epilepsy
EEG: Electroencephalogram
DG: Dentate gyrus
CA1: Cornu ammonis subfield 1
CA3: Cornu ammonis subfield 3
AP: Anteroposterior
ML: Mediolateral
DV: Dorsoventral
LFP: Local field potential
SLE: Seizure-like events
PBS: Phosphate buffered saline
NeuN: Neuronal nuclei
GFAP: Glial fibrillary acidic protein
Iba1: Ionized calcium binding adapter molecule 1
